# Prediction of pacemaker-induced cardiomyopathy using a convolutional neural network based on clinical findings prior to pacemaker implantation

**DOI:** 10.1038/s41598-024-57418-y

**Published:** 2024-03-22

**Authors:** Mitsunori Oida, Takuya Mizutani, Eriko Hasumi, Katsuhito Fujiu, Kosaku Goto, Kunihiro Kani, Tsukasa Oshima, Takumi J. Matsubara, Yu Shimizu, Gaku Oguri, Toshiya Kojima, Issei Komuro

**Affiliations:** 1 Department of Cardiovascular Medicine, Graduate School of Medicine, The University of Tokyo, 7-3-1 Hongo, Bunkyo, Tokyo, 113-8655 Japan; 2 Department of Advanced Cardiology, Graduate School of Medicine, The University of Tokyo, Tokyo, Japan; 3 Department of Radiology, Graduate School of Medicine, The University of Tokyo, Tokyo, Japan

**Keywords:** Cardiac device therapy, Heart failure

## Abstract

Risk factors for pacemaker-induced cardiomyopathy (PICM) have been previously reported, including a high burden of right ventricular pacing, lower left ventricular ejection fraction, a wide QRS duration, and left bundle branch block before pacemaker implantation (PMI). However, predicting the development of PICM remains challenging. This study aimed to use a convolutional neural network (CNN) model, based on clinical findings before PMI, to predict the development of PICM. Out of a total of 561 patients with dual-chamber PMI, 165 (mean age 71.6 years, 89 men [53.9%]) who underwent echocardiography both before and after dual-chamber PMI were enrolled. During a mean follow-up period of 1.7 years, 47 patients developed PICM. A CNN algorithm for prediction of the development of PICM was constructed based on a dataset prior to PMI that included 31 variables such as age, sex, body mass index, left ventricular ejection fraction, left ventricular end-diastolic diameter, left ventricular end-systolic diameter, left atrial diameter, severity of mitral regurgitation, severity of tricuspid regurgitation, ischemic heart disease, diabetes mellitus, hypertension, heart failure, New York Heart Association class, atrial fibrillation, the etiology of bradycardia (sick sinus syndrome or atrioventricular block) , right ventricular (RV) lead tip position (apex, septum, left bundle, His bundle, RV outflow tract), left bundle branch block, QRS duration, white blood cell count, haemoglobin, platelet count, serum total protein, albumin, aspartate transaminase, alanine transaminase, estimated glomerular filtration rate, sodium, potassium, C-reactive protein, and brain natriuretic peptide. The accuracy, sensitivity, specificity, and area under the curve of the CNN model were 75.8%, 55.6%, 83.3% and 0.78 respectively. The CNN model could accurately predict the development of PICM using clinical findings before PMI. This model could be useful for screening patients at risk of developing PICM, ensuring timely upgrades to physiological pacing to avoid missing the optimal intervention window.

## Introduction

Pacemaker implantation (PMI) is an indispensable therapy for patients with sick sinus syndrome (SSS) and atrioventricular block (AVB)^1^, and the number of patients receiving PMI has been increasing, with approximately one million devices now being implanted annually worldwide^2^. PMI-related deterioration of left ventricular (LV) systolic function is known as pacemaker-induced cardiomyopathy (PICM)^3^. The following three definitions of PICM have been used in past clinical studies: (a) left ventricular ejection fraction (LVEF) ≤ 40% if the baseline value is ≥ 50% or an absolute reduction in LVEF ≥ 5% if the baseline value is < 50%; (b) LVEF ≤ 40% if the baseline value is ≥ 50% or an absolute reduction in LVEF ≥ 10% if the baseline value is < 50%; and (c) absolute reduction in LVEF ≥ 10% regardless of the baseline value^4^.

Previous studies have identified several risk factors for PICM, including older age^5–7^, male sex^6^, a history of ischemic heart disease (IHD)^8,9^, a history of atrial fibrillation (AF)^5,10^, a history of chronic kidney disease^9^, lower baseline LVEF^11,12^, wider intrinsic QRS duration^6,13^, left bundle branch block (LBBB)^8^, and a chronic higher right ventricular (RV) pacing burden^14^. Upgrade to cardiac resynchronization therapy is often required in patients with PICM^15^. However, predicting the development of PICM is still challenging because the risk factors for PICM have not been well established^3^. Recently, machine learning techniques have been used increasingly in the medical field and are considered useful tools in daily practice. The aim of the present study was to develop a CNN that can predict the development of PICM before PMI using clinical findings prior to PMI.

## Methods

### Definition of PICM

PICM was defined based on previous reports as follows: (a) exclusion of an alternative cause of cardiomyopathy, such as de novo myocardial ischemia, uncontrollable tachyarrhythmia, frequent premature contractions, or untreated hypertension; and (b) a > 10% reduction in LVEF measured by transthoracic echocardiography (TTE) after PMI. The patients underwent TTE within the 6 months before PMI and between 3 months and 3 years after PMI.

### Data collection and study population

All data were retrospectively collected for the 561 patients identified to have undergone primary PMI for SSS or AVB at the University of Tokyo Hospital, Japan, between November 2006 and December 2021. The study inclusion criteria were as follows: age 20 years or over; primary PMI; and TTE data available both before and after PMI. The following exclusion criteria were applied: younger than 20 years; previous placement of a cardiac implantable electrical device; missing echocardiography data before and/or after PMI; history of heart transplantation; congenital heart disease; and an alternative cause of reduction in LVEF, such as de novo myocardial ischemia, uncontrollable tachyarrhythmia and frequent premature contractions, or untreated hypertension. Details of medical history and clinical data were retrospectively collected from all patients to identify variables that could predict the development of PICM. Their laboratory data were also obtained on admission to our hospital, and the results of follow-up TTE performed in the outpatient department.

**Figure 1:**
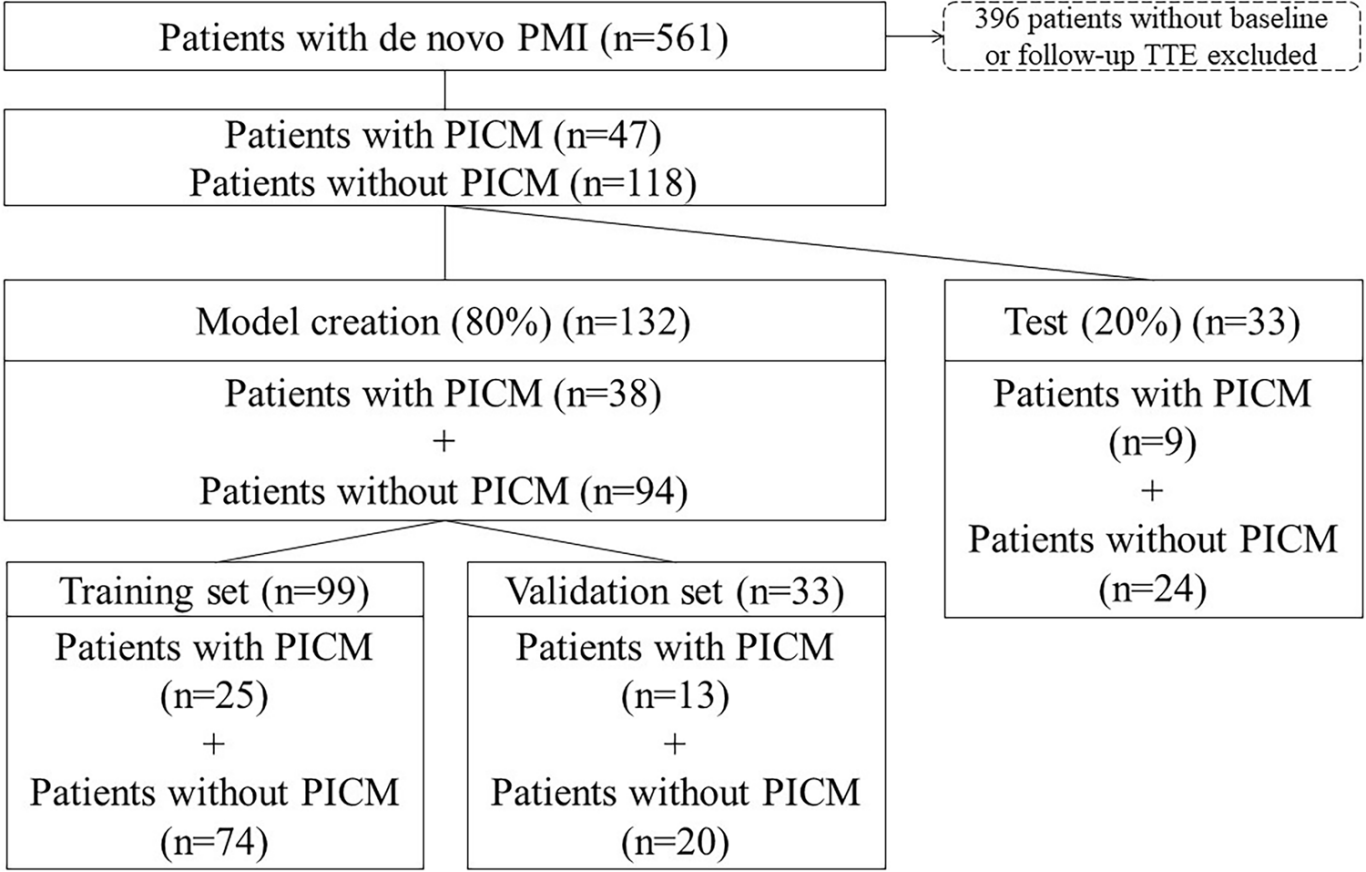
Flow chart showing the process used to collect data for the study population. The study investigated patients who underwent primary pacemaker implantation between December 2006 and December 2021. A total of 165 patients who met the criteria were enrolled in the study. PMI, pacemaker implantation; TTE, transthoracic echocardiogram; PICM, pacing-induced cardiomyopathy.

The clinical data for 165 patients (with PICM, n = 47; without PICM, n = 118) were divided into a training dataset (n = 99, 60%), a validation dataset (n = 33, 20%) and a test dataset (n = 33, 20%). The process used to collect the data for the study population is described in Fig. 1. Furthermore, due to the relatively small number of PICM patients in this study, we expanded the data using the Synthetic Minority Oversampling Technique (SMOTE) to ensure that PICM patients represented 50% of the total patient population.

### Definition of clinical variables and creation of the dataset

The clinical data included the following variables: age, sex, body mass index (BMI), LVEF, left ventricular end-diastolic diameter (LVEDd), left ventricular end-systolic diameter (LVEDs), left atrial diameter (LAD), and severity of mitral and tricuspid regurgitation (MR and TR, respectively; classified into trivial, mild, moderate, or severe by TTE before PMI), history of ischaemic heart disease (IHD, diagnosed by angiography or scintigraphy), diabetes mellitus (DM, defined as use of oral hypoglycaemic agents or insulin or a glycosylated haemoglobin of ≥ 6.5%), hypertension (HT, defined as use of antihypertensive agents, systolic blood pressure ≥ 140 mmHg, or diastolic blood pressure ≥ 90 mmHg), and heart failure (HF, defined as New York Heart Association [NYHA] class ≥ 2), NYHA class (categorized based on symptoms and assessment of the medical examination on admission by the cardiologists), history of AF (diagnosed by electrocardiogram), SSS or AVB (binodal disease was included in AVB), presenting with LBBB, QRS duration on electrocardiogram, RV lead tip position (divided into apex and non-apex), and laboratory data. The laboratory data included the following parameters: white blood cell count (WBC), haemoglobin (Hb), platelet count (Plt), serum total protein (TP), albumin (Alb), aspartate transaminase (AST), alanine transaminase (ALT), estimated glomerular filtration rate (eGFR, calculated as: 194 × (serum creatinine)^−1.094^ × (age)^−0.287^ × [0.739 for female patients]^16^), sodium (Na), potassium (K), C-reactive protein (CRP), and brain natriuretic peptide (BNP). NYHA class and severity of MR and TR ware treated as ordinal numeric variables.

Three convolutional neural network (CNN) model were constructed for predicting the development of PICM. Dataset 1 included the following 31 variables: age, sex, BMI, LVEF, LVEDd, LVEDs, LAD, severity of MR and TR, history of IHD, DM, HT, and HF, NYHA class, history of AF, the etiology of bradycardia (sick sinus syndrome [SSS] or atrioventricular block [AVB]), presenting with LBBB, QRS duration, RV lead tip position, WBC, Hb, Plt, TP, Alb, AST, ALT, eGFR, Na, K, CRP, and BNP. Dataset 2 included the following 10 variables: age, sex, BMI, LVEF, severity of TR, history of IHD, NYHA class, the etiology of bradycardia, eGFR, and CRP. Dataset 3 included the following 11 variables: age, sex, BMI, LVEF, severity of TR, history of IHD, NYHA class, the etiology of bradycardia, RV lead tip position, eGFR, and CRP (Table 1). Three CNN models (Model 1, 2, and 3) were made based on these datasets, respectively.

**Table 1:** Three types of datasets used for a convolutional neural network model for prediction of development of pacemaker-induced cardiomyopathy.

Dataset	Variables
1	Age, sex, body mass index, LVEF, LVEDd, LVEDs, left atrial diameter, severity of MR and TR, IHD, diabetes mellitus, hypertension, heart failure, NYHA class, AF, etiology of bradycardia (AVB or SSS), RV lead tip position (apex, septum, left bundle, His bundle, RV outflow tract), LBBB, QRS duration, WBC, haemoglobin, platelet count, serum total protein, albumin, AST, ALT, eGFR, Na, K, C-reactive protein, brain natriuretic peptide
2	Age, sex, body mass index, LVEF, severity of TR, IHD, NYHA class, indication for PMI, eGFR, C-reactive protein
3	Age, sex, body mass index, LVEF, severity of TR, IHD, NYHA class, indication for PMI, RV lead tip position (apex, septum, left bundle, His bundle, RV outflow tract), eGFR, C-reactive protein

*AF* atrial fibrillation; *AST* aspartate transaminase; *ALT* alanine transaminase; *AVB* atrioventricular block; *eGFR* estimated glomerular filtration rate; *IHD* ischaemic heart disease; *LBBB* left bundle branch block; *LVEDd* left ventricular end diastolic diameter; *LVEDs* left ventricular end systolic diameter; *LVEF* left ventricular ejection fraction; *MR* mitral regurgitation; *NYHA* New York Heart Association; *PMI* pacemaker implantation; *RV* right ventricular; *SSS* sick sinus syndrome; *TR* tricuspid regurgitation; *WBC* white blood cell count.

### Architecture of the CNN model

**Figure 2:**
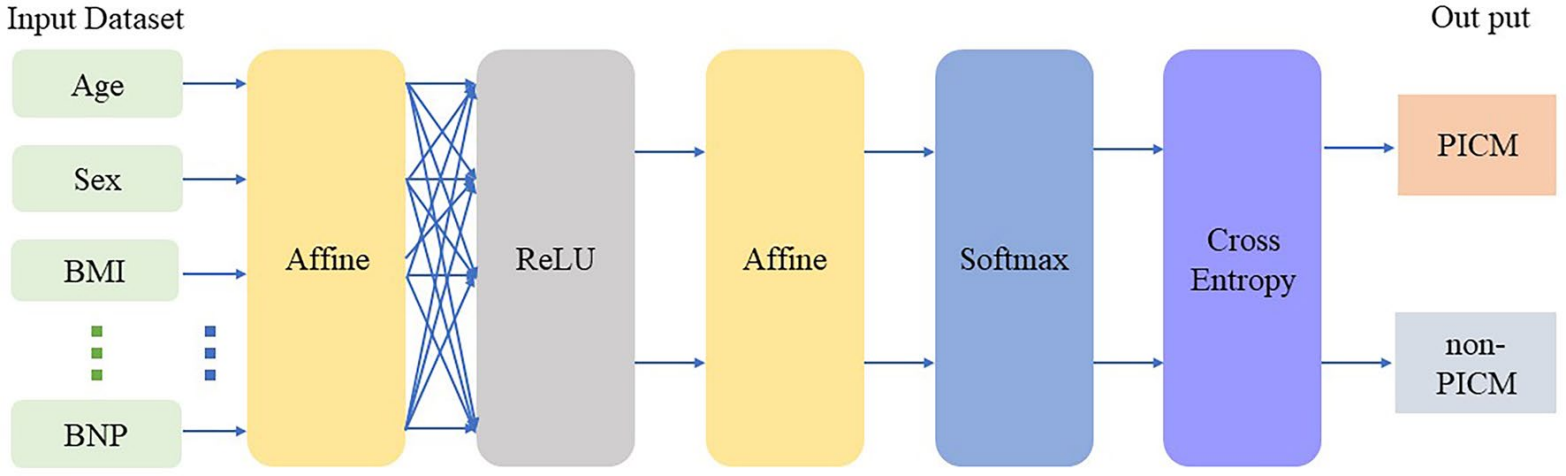
Neural network configuration. Construction of the convolutional neural network (CNN) consisted of the following layers: Input, Convolutional, Rectified Linear Unit (ReLU), Pooling, Fully Connected (Affine), Softmax, and Categorical Cross-Entropy. BMI, body mass index; BNP, brain natriuretic peptide; PICM, pacing-induced cardiomyopathy.

**Figure 3:**
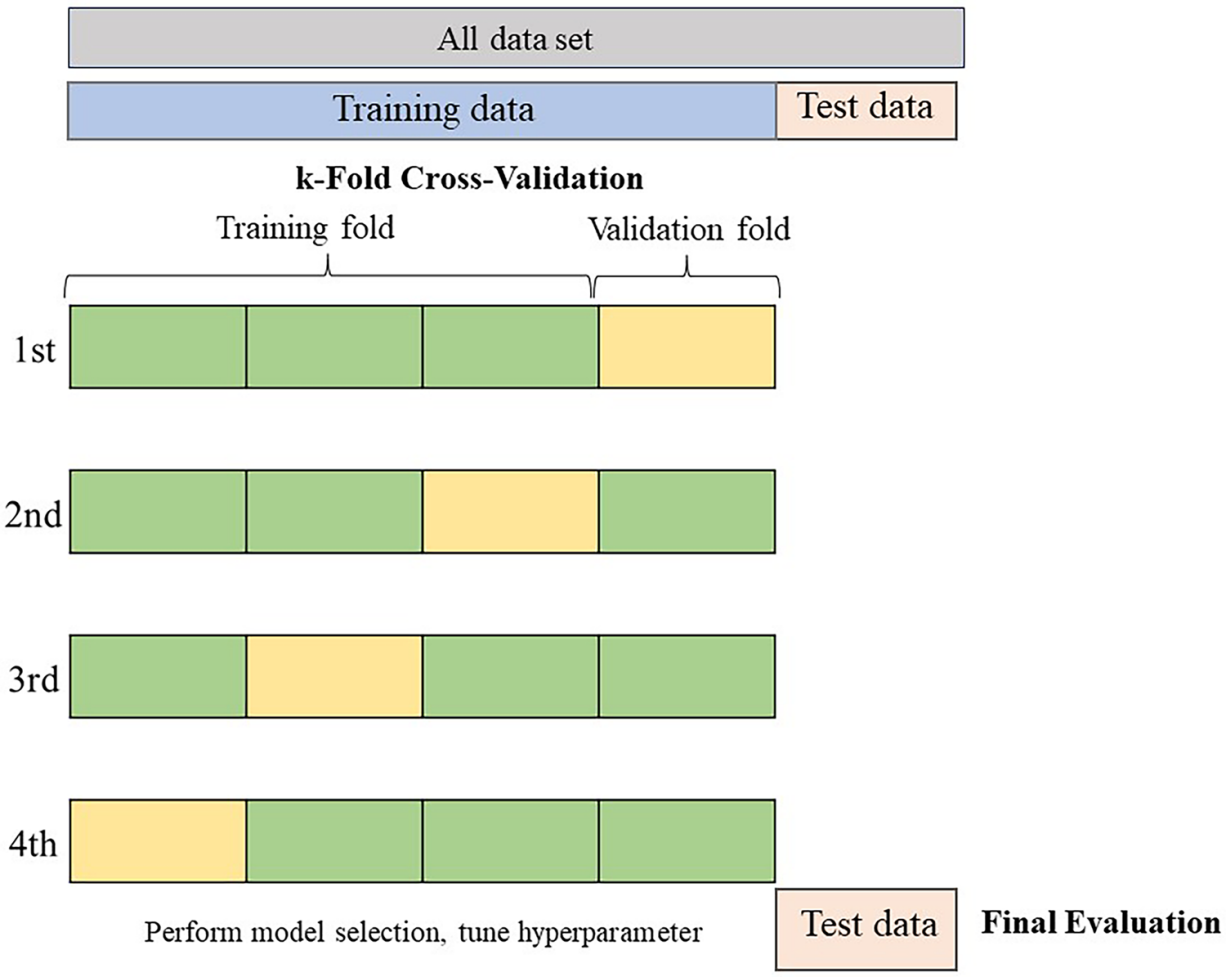
k-fold cross-validation. To mitigate overfitting, we designed a model that minimized the number of explanatory variables (regularization) and implemented k-fold cross-validation. We initially divided the training data into four subsets, conducting fourfold cross-validation. Each subset was used alternately as validation data, with the rest for training the model. This cycle was repeated to identify the training iteration count that minimized average loss across four trials. Using this optimal training iteration count, we retrained on the full training dataset and ultimately assessed the model's performance with test data.

We employed Python programming language and the Neural Network Console provided by Sony Corporation (Minato, Tokyo, Japan) for the construction of the Convolutional Neural Network (CNN) model. A graphic representation of the architecture is shown in Fig. 2. The k-fold cross validation method was used to improve the evaluation of CNN model, and k = 4 in this setting (Fig. 3).

### Evaluation

To evaluate the CNN model, the number of true positive, true negative, false positive, and false negative results were counted, and accuracy, sensitivity and specificity were calculated. Sensitivity and specificity were calculated according to “the closest-to-(0, 1) criterion”^17^. The predictive ability of the CNN model was evaluated using receiver-operating characteristic curve (ROC) analysis and the area under the curve (AUC). The 95% confidence intervals (95% CI) of AUCs were described. The Net Reclassification Improvement (NRI) metric was employed to assess the predictive ability of the three CNN models. To evaluate the contribution of variables in predicting PICM onset in three CNN models, we calculated the SHAP (SHapley Additive exPlanations) values for each variable.

### Statistical analysis

The patients were divided into a PICM group and a non-PICM group based on the previously described definition of PICM. Differences in baseline characteristics between the two groups were compared using Student’s *t*-test for continuous variables and the chi-squared test for categorical variables. DeLong's test was employed to compare the areas AUCs of three CNN models. All statistical analyses were performed using SPSS version 28.0 (IBM Corp., Armonk, NY, USA). A two-tailed *P* value < 0.05 was considered statistically significant.

### Ethical approval

This study was approved by the University of Tokyo institutional ethics committee (approval number 2650-13). For the retrospective cohort, all patient information was deidentified and the requirement for written informed consent was waived by the University of Tokyo institutional ethics committee. The study protocol was conducted in accordance with the Declaration of Helsinki.

## Results

### Characteristics of the study population

One hundred and sixty-five patients (89 men, 53.9%) who underwent primary PMI and had both pre-PMI and post-PMI TTE data available were enrolled in the study. During a mean follow-up of 1.7 ± 1.1 years, 47 patients (28.5%) developed PICM. The patient characteristics are shown in Table 2. Information on NYHA class, severity of MR/TR, and RV lead tip position are summarized in Supplementary Table S1. Patients who developed PICM had a significantly higher preimplantation LVEF (68.7 ± 13.3% vs. 63.5 ± 11.0%, *P* = 0.01) and were significantly more likely to have a history of IHD (51.1% vs. 28.0%, *P* < 0.01) and a lower eGFR (47.9 ± 24.7 mL/min/1.73 m^2^ vs. 61.8 ± 24.5 mL/min/1.73 m^2^, *P* < 0.01). There were no other statistically significant differences in variables between patients with and without PICM.

**Table 2:** Patient demographic and clinical characteristics.

Characteristic	Total (N = 165)	Non-PICM group (n = 118)	PICM group (n = 47)	*P* value
Demographic				
Age (years)	71.6 ± 11.2	71.8 ± 10.9	71.2 ± 12.1	0.68
Male sex, n (%)	89 (53.9)	61 (51.7)	28 (59.6)	0.36
Body mass index	22.6 ± 3.8	22.4 ± 3.8	23.1 ± 3.8	0.28
Echocardiographic				
LVEF (%)	65.0 ± 11.9	63.5 ± 11.0	68.7 ± 13.3	0.01***
LVEDd (mm)	46.5 ± 6.9	46.4 ± 6.8	46.4 ± 7.3	0.67
LVEDs (mm)	29.8 ± 7.0	30.2 ± 7.0	28.8 ± 6.9	0.25
LAD (mm)	41.6 ± 8.3	41.8 ± 8.5	41.3 ± 8.0	0.71
At least moderate MR, n (%)	9 (5.5)	6 (5.1)	3 (6.4)	0.72
At least moderate TR, n (%)	15 (9.1)	11 (9.3)	4 (8.5)	1.00
Medical history and clinical findings, n (%)				
IHD	57 (34.5)	33 (28.0)	24 (51.1)	< 0.01**
Diabetes mellitus	52 (31.5)	33 (28.0)	19 (40.4)	0.12
Hypertension	106 (64.2)	77 (65.3)	29 (61.7)	0.67
Heart failure	38 (23.0)	26 (22.0)	12 (25.5)	0.63
NYHA class ≥ II	78 (47.3)	55 (46.6)	23 (48.9)	0.79
Arrythmia and ECG findings				
AF, n (%)	64 (38.8)	48 (40.7)	16 (34.0)	0.43
AVB indicated PMI, n (%)	75 (45.5)	48 (40.7)	27 (57.4)	0.06
LBBB, n (%)	10 (6.1)	10 (8.5)	0 (0)	0.06
QRS duration (ms)	116.7 ± 26.6	117.8 ± 26.8	116.0 ± 26.3	0.84
RV lead tip position				
Apex, n (%)	42 (25.5)	32 (27.1)	10 (21.3)	0.44
Laboratory results				
WBC (× 10^3^/μL)	6.5 ± 2.3	6.4 ± 2.2	6.7 ± 2.5	0.44
Haemoglobin (g/dL)	12.3 ± 2.1	12.4 ± 2.0	12.1 ± 2.2	0.36
Platelet count (× 10^5^/μL)	21.7 ± 8.8	21.4 ± 9.0	22.2 ± 8.1	0.60
Total protein (g/dL)	6.5 ± 0.8	6.5 ± 0.8	6.5 ± 0.8	0.99
Albumin (g/dL)	3.7 ± 0.5	3.7 ± 0.5	3.7 ± 0.5	0.99
AST (IU/L)	42.3 ± 103.2	41.2 ± 89.3	23.3 ± 19.4	0.18
ALT (IU/L)	36.1 ± 76.5	44.2 ± 100.7	25.1 ± 21.1	0.18
eGFR (mL/min/1.73 m^2^)	57.8 ± 25.3	61.8 ± 24.5	47.90 ± 24.7	< 0.01**
Na (mmol/mL)	139.4 ± 3.1	139.6 ± 2.8	138.8 ± 3.7	0.14
K (mmol/mL)	4.4 ± 0.5	4.3 ± 0.4	4.4 ± 0.7	0.17
CRP (mg/dL)	1.0 ± 2.8	1.1 ± 3.1	0.7 ± 1.5	0.33
BNP (pg/mL)	268.1 ± 416.7	280.3 ± 423.3	300.6 ± 416.7	0.78

*AF* atrial fibrillation; *AST* aspartate transaminase; *ALT* alanine transaminase; *BNP* brain natriuretic peptide; *eGFR* estimated glomerular filtration rate; *IHD* ischaemic heart disease; *LBBB* left bundle branch block; *LVEDd* left ventricular end diastolic diameter; *LVEDs* left ventricular end systolic diameter; *LVEF* left ventricular ejection fraction; *MCV* mean corpuscular volume; *MR* mitral regurgitation; *NYHA* New York Heart Association; *PICM* pacemaker-induced cardiomyopathy; *PMI* pacemaker implantation; *RV* right ventricular; *TR* tricuspid regurgitation; *WBC* white blood cell count. **P < 0.01.

No statistically significant difference in baseline characteristics was detected among patients in the training, validation, and test datasets (Supplementary Table S2). Moreover, there were no statistically significant differences between the four-fold cross-validation datasets and the test dataset (Supplementary Table S3).

### Evaluation of the machine learning model for predicting the development of PICM

**Figure 4:**
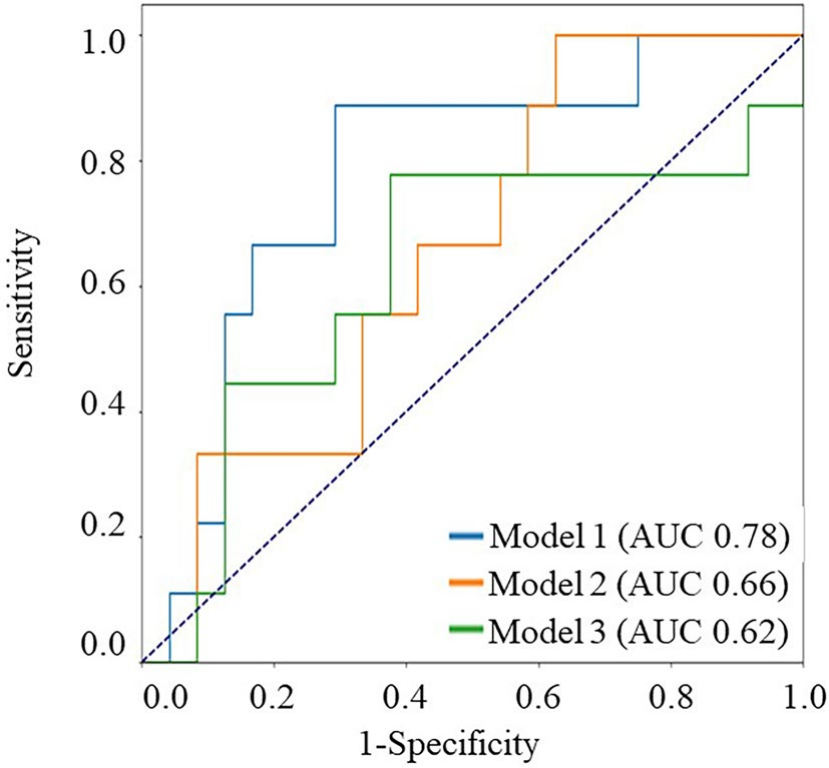
Receiver-operating characteristic curves for three models. A receiver-operating characteristic curve connects coordinate points with the false positive rate (1—specificity) on the x-axis and sensitivity on the y-axis, calculated from the test results at various cut-off values. Among the three models, Model 1 achieved the highest accuracy, with an AUC of 0.78. AUC, area under the curve.

The accuracy, sensitivity, and specificity of each model are shown in Table 3. Receiver-operating characteristic curves are shown in Fig. 4, and the area under the curve for each model was 0.78 (0.59–0.95), 0.66 (0.45–0.86), and 0.62 (0.36–0.86), respectively. The variables selected to construct each three model and SHAP value of these are shown in Supplemental Figure S1. Based on the SHAP values, variables such as the etiology of bradycardia, eGFR and IHD were more important for predicting the onset of PICM in all CNN models. Conversely, factors previously identified as risk factors for the onset of PICM in cohort studies, such as LVEF, LBBB, and QRS duration, did not contribute significantly to Model 1, which demonstrated the highest accuracy in predicting the onset of PICM.

**Table 3:** Accuracy of the three CNN models in predicting onset of pacemaker-induced cardiomyopathy.

	Accuracy (%)	Sn (%)	Sp (%)	AUC	*P* value (Delong’s test)	NRI (%)
Model 1	75.8	55.6	83.3	0.78 (0.59–0.95)	N/A	N/A
Model 2	57.6	33.3	66.7	0.66 (0.45–0.86)	< 0.01**	− 38.89
Model 3	63.6	55.6	66.7	0.62 (0.36–0.86)	< 0.01**	− 16.67

*Sn* sensitivity; *Sp* specificity; *AUC* area under the curve; *NRI* net reclassification improvement; *N/A* not applicable. **P < 0.01.

## Discussion

In this study, the prevalence of the development of PICM was consistent with that previously reported^18^. We developed a CNN for prediction of PICM using three types of datasets as described in Table 1. Variables in Dataset 1 included factors previously reported as risk factors for the onset of PICM^5–12,14^. Among the three models evaluated, Model 1, which incorporated the largest number of these variables, achieved the highest specificity.

In our study, our CNN algorithm exclusively employed numerical data as a contributing factor and did not incorporate image information. Consequently, there are situations where classical machine learning methods could offer certain advantages. Nevertheless, in our pursuit of greater precision, we selected to implement a CNN model. We conducted a comparative analysis with classical machine learning models, and the CNN model consistently demonstrated the highest accuracy. These results are presented in Table S4 within the Supplemental Data.

The variables used to construct Model 1, which showed the highest accuracy of all the models, are obtainable in daily practice. The CNN we have developed enabled us to predict the risk of PICM with some clinical information available before PMI, making it feasible for clinical practice. This CNN model has the potential to assist in identifying patients with PMI who require more intensive management, ensuring that timely upgrades to biventricular pacing/defibrillation systems are not overlooked.

However, this study suggests that although models incorporating multiple variables tend to yield higher prediction accuracy, obtaining comprehensive clinical information can be challenging in daily practice. Given this context, there is a need for predicting PICM using minimal clinical information. In this research, due to the limited patient sample size, the model encompassing the greatest number of variables demonstrated superior accuracy. Nonetheless, with an increase in patient numbers, it may become feasible to refine the model, potentially altering the significance of each variable. This could ultimately lead to a reduction in the required parameters, thereby facilitating the development of a more adaptable CNN model.

Our created model has a relatively lower sensitivity in diagnosing PICM, but the specificity is relatively high at 83.3%. In the context of predicting the onset of a condition, this specificity is not necessarily low. For instance, recently, other deep learning models predicting the onset of AF from electrocardiogram data have reported accuracies with AUC values ranging from 0.71 to 0.82^19^, and the best AUC in our study of 0.78 was comparable. Furthermore, it is important to consider that the role of our artificial intelligence (AI) model is not to identify patients who could develop the condition from the general population, but rather to differentiate patients at a lower risk of developing PICM. This is especially relevant for patients with implanted pacemakers who generally have normal cardiac function and primarily require periodic pacemaker interrogations. For these patients, if the AI assesses them as having a lower risk of developing PICM, their routine pacemaker check-ups will be continued as usual. On the other hands, for other patients, conducting regular examination, including consultations and TTE, annually or biannually can be recommended. This approach could encourage to eliminate unnecessary tests, while effectively identifying high-risk patients.

This study had several limitations. First, it was a retrospective analysis conducted at a single tertiary care centre, which may limit the generalizability of its findings. Some biases such as selection bias and observer bias should be considered. Some patients with PMI were excluded due to unmatched to inclusion criteria. Moreover, inter- and intra-observer variabilities in TTE were not assessed. Consequently, it remains unclear these variabilities could have influenced the results. Second, the procedural protocol for RV lead placement in our study was not standardized; that is, various pacing sites were used because of the recent prevailing conduction system pacing strategies. Therefore, further studies that include larger sample sizes and in-depth clinical studies are needed to improve the accuracy and confirm the feasibility of our CNN model. Finally, although our CNN model predicts for the occurrence of PICM based on pre-implantation information, our model is uncapable to predict the time of onset for the development of PICM. Previous reports on the occurring PICM ranges from 1 month^20^ to 16.9 years^6^ after PMI, showing significant variation, and there is currently no clear consensus of its onset. Therefore, it is challenging to determine when and how often postoperative TTE should be performed. In some patients, PICM may develop a long time after PMI, thus it is recommended to conduct regular examinations every six months to a year. The development of AI models capable of predicting the probability and time of PICM occurrence in patients with pacemaker could enable more accurate screening for those requiring regular examination.

In conclusion, we have demonstrated the potential of utilizing a CNN with available clinical information to predict development of PICM before PMI. Clinicians in daily practice can utilize a CNN to identify patients who are at risk of developing PICM, which has the potential to prevent overlooking timely upgrades to biventricular pacing systems.

### Supplementary Information

Supplementary Information.

## Data Availability

The datasets generated and analyzed during the current study are not publicly available due to privacy reasons but are available from the corresponding author on reasonable request.
