# Supplementary material for: Prediction of pacemaker-induced cardiomyopathy using a convolutional neural network based on clinical findings prior to pacemaker implantation
Source: Sci Rep. 2024 Mar 22;14:6916. doi: 10.1038/s41598-024-57418-y (PMC10959989; doi:10.1038/s41598-024-57418-y)
Supplement: Supplementary file 1 — Supplementary Information. [file 41598_2024_57418_MOESM1_ESM.docx]

## Neural Network Configuration

For this analysis, we employed several layers, including Input, Affine, Rectified Linear Unit (ReLU), Affine2, and Softmax. The Affine function served as the all-attachment layer, combining all input values to all output layers as determined by the out-shape property. The ReLU function was applied such that when the input value was zero or less, the output value was set to zero, and when the input value exceeded zero, the output value matched the input value. The Softmax function converted the cumulative output values into 1.0, ensuring that each output value was always within the range of 0.0 to 1.0, allowing these values to be interpreted as probabilities. Finally, the loss function, Categorical Cross-Entropy, was employed at the output layer of the neural network, aiming to minimize the cross-entropy with respect to the variables in the dataset provided by the input layer (Figure 2).

## Hyperparameter Optimization

To reduce overfitting, we designed a model that minimizes the number of explanatory variables (regularization) and implemented k-fold cross-validation. For hyperparameter optimization, we used Grid search. We focused on three parameters in the neural network: number of layers, number of nodes, and number of training iterations. We first split the training data into four subsets and performed 4-fold cross-validation. Each subset was used alternately as validation data, and the rest were used to train the model. We repeated this cycle to determine the number of training iterations that minimized the average loss over four trials. Using this optimal number of training iterations, we retrained on the full training dataset and finally evaluated the performance of the model using test data. The optimal combination of “number of layers” and “number of nodes” was selected based on four evaluation metrics: accuracy, sensitivity, specificity, and area under the curve. Considering the performance evaluation heat map based on the layers and number of nodes of the neural network model shown below, the number of layers was set to 11 and the number of nodes was set to 64.

**Supplementary Table S1.**

**Comparison of baseline characteristics between patients with and without pacemaker-induced cardiomyopathy**

| **Characteristic** | **Total (n=165)** | **Non-PICM group (n=118)** | **PICM group**  **(n=47)** | **P-value** |
| --- | --- | --- | --- | --- |
| **NYHA class, n (%)** |  |  |  | 0.34 |
| I | 87 (52.7) | 63 (53.4) | 24 (51.1) |  |
| II | 52 (31.5) | 38 (32.2) | 14 (29.8) |  |
| III | 22 (13.3) | 13 (11.0) | 9 (19.1) |  |
| IV | 4 (2.4) | 4 (3.4) | 0 (0) |  |
| **Severity of MR, n (%)** |  |  |  | 0.69 |
| Trivial | 89 (53.9) | 64 (54.3) | 25 (53.2) |  |
| Mild | 67 (40.6) | 48 (40.7) | 19 (40.4) |  |
| Moderate | 7 (4.2) | 5 (4.2) | 2 (4.3) |  |
| Severe | 2 (1.2) | 1 (0.8) | 1 (2.1) |  |
| **Severity of TR, n (%)** |  |  |  | 0.65 |
| Trivial at most | 74 (44.9) | 50 (42.3) | 24 (51.0) |  |
| Mild | 76 (46.1) | 57 (48.3) | 19 (40.4) |  |
| Moderate | 13 (7.9) | 9 (7.6) | 4 (8.5) |  |
| Severe | 2 (1.7) | 2 (1.2) | 0 (0) |  |
| **RV lead tip position, n (%)** |  |  |  | 0.93 |
| Apex | 42 (25.5) | 32 (27.1) | 10 (21.3) |  |
| Septum | 97 (58.8) | 67 (56.8) | 30 (63.8) |  |
| Left bundle | 12 (7.3) | 9 (7.6) | 3 (6.4) |  |
| His bundle | 10 (6.1) | 7 (5.9) | 3 (6.4) |  |
| RVOT | 4 (2.4) | 3 (2.5) | 1 (2.1) |  |

MR, mitral regurgitation; NYHA, New York Heart Association; PICM, pacemaker-induced cardiomyopathy; RVOT, right ventricular outflow tract; TR, tricuspid regurgitation

**Supplementary Table S2.**

**Comparison of characteristics between patients in the training, validation, and test datasets**

| **Characteristics** | **Total**  **(N=165)** | **Training set**  **(n=99)** | **Validation set**  **(n=33)** | **Test set**  **(n=33)** | ***P*-value** |
| --- | --- | --- | --- | --- | --- |
| **Demographic** |  |  |  |  |  |
| Age (years) | 71.6 ± 11.2 | 72.4 ± 10.9 | 70.2 ± 10.9 | 70.5 ± 12.5 | 0.52 |
| Male sex, n, %) | 89 (53.9) | 52 (52.5) | 21 (63.6) | 16 (48.5) | 0.42 |
| Body mass index | 22.6 ± 3.8 | 22.8 ± 3.5 | 22.5 ± 4.5 | 22.0 ± 3.9 | 0.50 |
| **Echocardiographic** |  |  |  |  |  |
| LVEF, % | 65.0 ± 11.9 | 65.8 ± 11.5 | 64.1 ± 13.2 | 63.7 ± 11.9 | 0.62 |
| LVEDd (mm) | 46.5 ± 6.9 | 46.7 ± 6.9 | 46.3 ± 7.9 | 46.3 ± 6.2 | 0.95 |
| LVEDs (mm) | 29.8 ± 7.0 | 29.7 ± 6.9 | 29.5 ± 7.6 | 30.2 ± 6.6 | 0.92 |
| LAD (mm) | 41.6 ± 8.3 | 41.6 ± 8.4 | 40.9 ± 8.3 | 42.6 ± 8.1 | 0.69 |
| **Severity of MR, n (%)** |  |  |  |  | 0.08 |
| Trivial at most | 89 (53.9) | 53 (53.5) | 24 (72.7) | 12 (36.3) |  |
| Mild | 67 (40.6) | 42 (42.4) | 6 (18.2) | 19 (57.6) |  |
| Moderate | 7 (4.2) | 3 (3.0) | 2 (6.1) | 2 (6.1) |  |
| Severe | 2 (1.2) | 1 (1.0) | 1 (3.0) | 0 (0) |  |
| **Severity of TR, n (%)** |  |  |  |  | 0.49 |
| Trivial at most | 74 (44.9) | 43 (43.4) | 17 (51.5) | 14 (42.5) |  |
| Mild | 76 (46.1) | 47 (47.5) | 14 (42.4) | 15 (45.5) |  |
| Moderate | 13 (7.9) | 8 (8.1) | 2 (6.1) | 3 (9.1) |  |
| Severe | 2 (1.7) | 1 (1.0) | 0 (0) | 1 (3.0) |  |
| **Medical history and clinical findings, n (%)** |  |  |  |  |  |
| IHD | 57 (34.5) | 32 (32.3) | 16 (48.5) | 9 (27.3) | 0.15 |
| Diabetes mellitus | 52 (31.5) | 31 (31.3) | 11 (33.3) | 10 (30.3) | 0.96 |
| Hypertension | 106 (64.2) | 64 (64.6) | 19 (57.6) | 23 (69.7) | 0.59 |
| Heart failure | 38 (23.0) | 23 (23.2) | 6 (18.2) | 9 (27.3) | 0.68 |
| **NYHA class, n (%)** |  |  |  |  | 0.94 |
| I | 87 (52.7) | 52 (52.5) | 19 (57.6) | 16 (48.5) |  |
| II | 52 (31.5) | 32 (32.3) | 9 (27.3) | 11 (33.3) |  |
| III | 22 (13.3) | 12 (12.1) | 5 (15.2) | 5 (15.2) |  |
| IV | 4 (2.4) | 3 (3.0) | 0 (0) | 1 (3.0) |  |
| PICM | 47 (28.5) | 25 (25.3) | 13 (39.4) | 9 (27.3) | 0.29 |
| **Arrythmia and ECG findings** |  |  |  |  |  |
| Atrial fibrillation, n (%) | 64 (38.8) | 39 (39.4) | 13 (39.4) | 12 (36.4) | 0.95 |
| AVB indication for PMI, n (%) | 75 (45.5) | 42 (42.4) | 15 (45.5) | 18 (54.5) | 0.48 |
| LBBB, n (%) | 10 (6.1) | 7 (7.1) | 1 (3.0) | 2 (6.1) | 0.70 |
| QRS duration (ms) | 116.7 ± 26.6 | 118.2 ± 28.5 | 113.9 ± 25.3 | 115.1 ± 22.3 | 0.67 |
| **RV lead tip position, n (%)**  Apex (vs. non-apex) | 42 (25.5) | 29 (29.3) | 5 (15.2) | 8 (24.2) | 0.27 |
| Apex | 42 (25.5) | 29 (29.3) | 5 (15.2) | 8 (24.2) | 0.79 |
| Septum | 97 (58.8) | 57 (57.6) | 22 (66.7) | 18 (54.5) |  |
| Left bundle | 12 (7.3) | 6 (6.1) | 2 (6.1) | 4 (12.1) |  |
| His bundle | 10 (6.1) | 6 (6.1) | 3 (9.1) | 1 (3.0) |  |
| RVOT | 4 (2.4) | 2 (2.0) | 1 (3.0) | 1 (3.0) |  |
| **Laboratory results** |  |  |  |  |  |
| WBC (×10^3^/μL) | 6.5 ± 2.3 | 6.6 ± 2.4 | 6.4 ± 2.3 | 6.5 ± 1.7 | 0.89 |
| Haemoglobin (g/dL) | 12.3 ± 2.1 | 12.3 ± 2.1 | 12.4 ± 2.2 | 12.3 ± 2.0 | 0.97 |
| Platelets (×10^5^/μL) | 21.7 ± 8.8 | 21.0 ± 8.9 | 24.1 ± 9.1 | 21.2 ± 7.9 | 0.21 |
| Total protein (g/dL) | 6.5 ± 0.8 | 6.5 ± 0.7 | 6.7 ± 1.1 | 6.5 ± 0.8 | 0.68 |
| Albumin (g/dL) | 3.7 ± 0.5 | 3.7 ± 0.5 | 3.8 ± 0.5 | 3.8 ± 0.5 | 0.39 |
| AST (IU/L) | 42.3 ± 103.2 | 46.1 ± 117.7 | 28.4 ± 21.6 | 44.7 ± 107.2 | 0.69 |
| ALT (IU/L) | 36.1 ± 76.5 | 40.1 ± 86.5 | 25.7 ± 25.8 | 34.3 ± 79.2 | 0.64 |
| eGFR (mL/min/1.73 m^2^) | 57.8 ± 25.3 | 55.7 ± 23.5 | 64.6 ± 24.7 | 57.3 ± 30.1 | 0.22 |
| Na (mmol/mL) | 139.4 ± 3.1 | 139.4 ± 3.2 | 139.7 ± 2.7 | 139.0 ± 3.3 | 0.66 |
| K (mmol/mL) | 4.4 ± 0.5 | 4.4 ± 0.6 | 4.3 ± 0.5 | 4.4 ± 0.4 | 0.42 |
| C-reactive protein (mg/dL) | 1.0 ± 2.8 | 1.1 ± 3.3 | 0.8 ± 1.6 | 1.0 ± 1.8 | 0.85 |
| BNP (pg/mL) | 268.1 ± 416.7 | 332.4 ± 484.0 | 194.1 ± 325.2 | 239.2 ± 221.5 | 0.20 |

ALT, alanine transaminase; AST, aspartate transaminase; AVB, atrioventricular block; BNP, brain natriuretic peptide; ECG, electrocardiogram; eGFR, estimated glomerular filtration rate; IHD, ischaemic heart disease; LAD, left atrial diameter; LBBB, left bundle branch block; LVEDd, left ventricular end diastolic diameter; LVEDs, left ventricular end systolic diameter; LVEF, left ventricular ejection fraction; MCV, mean corpuscular volume; MR, mitral regurgitation; NYHA, New York Heart Association; PICM, pacemaker-induced cardiomyopathy; PMI, pacemaker implantation; TR, tricuspid regurgitation; WBC, white blood cell count

**Supplementary Table S3.**

**Comparison of characteristics between patients in the four-fold datasets and the test dataset**

| **Characteristics** | **Total** | **Fold #1** | **Fold #2** | **Fold #3** | **Fold #4** | **Test** | ***P*-value** |
| --- | --- | --- | --- | --- | --- | --- | --- |
|  | **(N=165)** | **(n=33)** | **(n=33)** | **(n=33)** | **(n=33)** | **(n=33)** |  |
| **Demographic** |  |  |  |  |  |  |  |
| Age (years) | 71.6 ± 11.2 | 69.5 ± 15.0 | 74.6 ± 6.8 | 73.1 ± 8.9 | 70.2 ± 10.9 | 70.5 ± 12.5 | 0.30 |
| Male sex (n, %) | 89 (53.9) | 19 (57.6) | 17 (51.5) | 16 (48.5) | 21 (63.6) | 16 (48.5) | 0.68 |
| Body mass index | 22.6 ± 3.8 | 22.5 ± 3.1 | 23.6 ± 3.9 | 22.6 ± 3.4 | 22.5 ± 4.5 | 22.0 ± 3.9 | 0.53 |
|  |  |  |  |  |  |  |  |
| **Echocardiographic** |  |  |  |  |  |  |  |
| LVEF (%) | 65.0 ± 11.9 | 63.6 ± 13.2 | 66.8 ± 9.3 | 66.8 ± 11.8 | 64.1 ± 13.2 | 63.7 ± 11.9 | 0.65 |
| LVEDd (mm) | 46.5 ± 6.9 | 47.0 ± 6.5 | 46.8 ± 5.8 | 46.2 ± 8.3 | 46.3 ± 7.9 | 46.3 ± 6.2 | 0.99 |
| LVEDs (mm) | 29.8 ± 7.0 | 30.4 ± 7.0 | 29.3 ± 6.5 | 29.5 ± 7.5 | 29.5 ± 7.6 | 30.2 ± 6.6 | 0.96 |
| LAD (mm) | 41.6 ± 8.3 | 40.5 ± 7.9 | 42.5 ± 10.0 | 41.7 ± 7.3 | 40.9 ± 8.3 | 42.6 ± 8.1 | 0.78 |
| **Severity of MR, n (%)** |  |  |  |  |  |  | 0.23 |
| Trivial at most | 89 (53.9) | 19 (57.6) | 17 (51.5) | 53 (53.5) | 24 (72.7) | 12 (36.3) |  |
| Mild | 67 (40.6) | 11 (33.3) | 16 (48.5) | 15 (45.5) | 6 (18.2) | 19 (57.6) |  |
| Moderate | 7 (4.2) | 2 (6.1) | 0 (0) | 1 (3.0) | 2 (6.1) | 2 (6.1) |  |
| Severe | 2 (1.2) | 1 (3.0) | 0 (0) | 0 (0) | 1 (3.0) | 0 (0) |  |
| **Severity of TR, n (%)** |  |  |  |  |  |  | 0.62 |
| Trivial at most | 74 (44.9) | 16 (48.5) | 11 (33.3) | 16 (48.5) | 17 (51.5) | 14 (42.5) |  |
| Mild | 76 (46.1) | 13 (39.4) | 19 (57.6) | 15 (45.5) | 14 (42.4) | 15 (45.5) |  |
| Moderate | 13 (7.9) | 3 (9.1) | 3 (9.1) | 2 (6.1) | 2 (6.1) | 3 (9.1) |  |
| Severe | 2 (1.7) | 1 (3.0) | 0 (0) | 0 (0) | 0 (0) | 1 (3.0) |  |
| **Medical history and clinical findings, n (%)** |  |  |  |  |  |  |  |
| IHD | 57 (34.5) | 9 (27.3) | 9 (27.3) | 14 (42.4) | 16 (48.5) | 9 (27.3) | 0.19 |
| Diabetes mellitus | 52 (31.5) | 10 (30.3) | 10 (30.3) | 10 (33.3) | 11 (33.3) | 10 (30.3) | 1.00 |
| Hypertension | 106 (64.2) | 16 (48.5) | 26 (78.8) | 22 (66.7) | 19 (57.6) | 23 (69.7) | 0.10 |
| Heart failure | 38 (23.0) | 7 (21.2) | 8 (24.2) | 8 (24.2) | 6 (18.2) | 9 (27.3) | 0.93 |
| **NYHA class, n (%)** |  |  |  |  |  |  | 0.91 |
| I | 87 (52.7) | 20 (60.6) | 18 (54.5) | 14 (42.4) | 19 (57.6) | 16 (48.5) |  |
| II | 52 (31.5) | 10 (30.3) | 11 (30.3) | 12 (36.4) | 9 (27.3) | 11 (33.3) |  |
| III | 22 (13.3) | 3 (9.1) | 4 (12.1) | 5 (15.2) | 5 (15.2) | 5 (15.2) |  |
| IV | 4 (2.4) | 0 (0) | 1 (3.0) | 2 (6.1) | 0 (0) | 1 (3.0) |  |
| PICM | 47 (28.5) | 9 (27.3) | 7 (21.2) | 9 (27.3) | 13 (39.4) | 9 (27.3) | 0.58 |
| **Arrythmia and ECG findings** |  |  |  |  |  |  |  |
| AF, n (%) | 64 (38.8) | 10 (30.3) | 19 (57.6) | 10 (30.3) | 13 (39.4) | 12 (36.4) | 0.14 |
| AVB indication for PMI, n, (%) | 75 (45.5) | 16 (48.5) | 11 (33.3) | 15 (45.5) | 15 (45.5) | 18 (54.5) | 0.53 |
| LBBB, n (%) | 10 (6.1) | 1 (3.0) | 3 (9.1) | 3 (9.1) | 1 (3.0) | 2 (6.1) | 0.71 |
| QRS duration (ms) | 116.7 ± 26.6 | 122.2 ± 26.9 | 116.8 ± 30.2 | 115.6 ± 28.6 | 113.9 ± 25.3 | 115.1 ± 22.3 | 0.75 |
| **RV lead tip position, n (%)** |  |  |  |  |  |  | 0.27 |
| Apex (vs. non-apex) | 42 (25.5) | 8 (24.2) | 7 (21.2) | 14 (42.4) | 5 (15.2) | 8 (24.2) | 0.13 |
| Apex | 42 (25.5) |  |  | 29 (29.3) | 5 (15.2) | 8 (24.2) | 0.79 |
| Septum | 97 (58.8) |  |  | 57 (57.6) | 22 (66.7) | 18 (54.5) |  |
| Left bundle | 12 (7.3) |  |  | 6 (6.1) | 2 (6.1) | 4 (12.1) |  |
| His bundle | 10 (6.1) |  |  | 6 (6.1) | 3 (9.1) | 1 (3.0) |  |
| RVOT | 4 (2.4) |  |  | 2 (2.0) | 1 (3.0) | 1 (3.0) |  |
| **Laboratory results** |  |  |  |  |  |  |  |
| WBC (×10^3^/μL) | 6.5 ± 2.3 | 7.0 ± 3.2 | 6.1 ± 2.2 | 6.7 ± 1.6 | 6.4 ± 2.3 | 6.5 ± 1.7 | 0.55 |
| Haemoglobin (g/dL) | 12.3 ± 2.1 | 12.5 ± 2.2 | 12.3 ± 2.2 | 12.1 ± 2.4 | 12.4 ± 2.2 | 12.3 ± 2.0 | 0.96 |
| Platelets (×10^5^ /μL) | 21.7 ± 8.8 | 19.4 ± 5.9 | 22.2 ± 7.5 | 21.4 ± 12.1 | 24.1 ± 9.1 | 21.2 ± 7.9 | 0.31 |
| Total protein (g/dL) | 6.5 ± 0.8 | 6.4 ± 0.8 | 6.7 ± 0.6 | 6.4 ± 0.7 | 6.7 ± 1.1 | 6.5 ± 0.8 | 0.55 |
| Albumin (g/dL) | 3.7 ± 0.5 | 3.7 ± 0.7 | 3.8 ± 0.4 | 3.6 ± 0.5 | 3.8 ± 0.5 | 3.8 ± 0.5 | 0.18 |
| AST (IU/L) | 42.3 ± 103.2 | 48.2 ± 130.9 | 29.6 ± 19.0 | 60.6 ± 156.2 | 28.4 ± 21.6 | 44.7 ± 107.2 | 0.69 |
| ALT (IU/L) | 36.1 ± 76.5 | 46.2 ± 122.4 | 31.1 ± 31.9 | 43.1 ± 82.2 | 25.7 ± 25.8 | 34.3 ± 79.2 | 0.81 |
| eGFR (mL/min/1.73 m^2^) | 57.8 ± 25.3 | 57.7 ± 25.2 | 56.9 ± 21.8 | 52.5 ± 23.9 | 64.6 ± 24.7 | 57.3 ± 30.1 | 0.43 |
| Na (mmol/mL) | 139.4 ± 3.1 | 140.3 ± 2.5 | 138.8 ± 3.5 | 139.0 ± 3.3 | 139.7 ± 2.7 | 139.0 ± 3.3 | 0.22 |
| K (mmol/mL) | 4.4 ± 0.5 | 4.4 ± 0.4 | 4.4 ± 0.7 | 4.5 ± 0.6 | 4.3 ± 0.5 | 4.4 ± 0.4 | 0.65 |
| CRP (mg/dL) | 1.0 ± 2.8 | 1.5 ± 5.3 | 0.4 ± 0.6 | 1.4 ± 2.1 | 0.8 ± 1.6 | 1.0 ± 1.8 | 0.46 |
| BNP (pg/mL) | 268.1 ± 416.7 | 341.8 ± 473.1 | 218.5 ± 205.0 | 437.0 ± 653.3 | 194.1 ± 325.2 | 239.2 ± 221.5 | 0.10 |

AF, atrial fibrillation; ALT, alanine transaminase; AST, aspartate transaminase; AVB, atrioventricular block; BNP, brain natriuretic peptide; CRP, C-reactive protein; ECG, electrocardiogram; eGFR, estimated glomerular filtration rate; IHD, ischaemic heart disease; LAD, left atrium diameter; LBBB, left bundle branch block; LVEDd, left ventricular end diastolic diameter; LVEDs, left ventricular end systolic diameter; LVEF, left ventricular ejection fraction; MCV, mean corpuscular volume; MR, mitral regurgitation; NYHA, New York Heart Association class; PICM, pacemaker-induced cardiomyopathy; PMI, pacemaker implantation; RVOT, right ventricular outflow tract; TR, tricuspid regurgitation; WBC, white blood cell count

**Supplementary Table S4.**

**Comparison of Performance between Classical Machine Learning and Deep Learning Methods for Predicting Pacemaker-Induced Cardiomyopathy**

| Model 1 | Classifier | Accuracy (%) | Sn (%) | Sp (%) | AUC | *P*-value |
| --- | --- | --- | --- | --- | --- | --- |
|  | **CNN** | **75.8** | **55.6** | **83.3** | **0.78 (0.59–0.95)** | **N/A** |
|  | SVM | 54.5 | 55.6 | 54.2 | 0.53 (0.25–0.79) | < 0.01** |
|  | LR | 57.6 | 44.4 | 62.5 | 0.57 (0.34–0.80) | < 0.01** |
|  | LDA | 51.5 | 44.4 | 54.2 | 0.56 (0.34–0.77) | < 0.01** |
|  | KNN | 42.4 | 55.6 | 37.5 | 0.44 (0.19–0.68) | < 0.01** |
|  | RF | 57.6 | 11.1 | 75.0 | 0.44 (0.19–0.69) | < 0.01** |
|  | DT | 54.5 | 44.4 | 58.3 | 0.51 (0.32–0.72) | < 0.01** |
| Model 2 | Classifier | Accuracy (%) | Sn (%) | Sp (%) | AUC | *P*-value |
|  | CNN | 57.6 | 33.3 | 66.7 | 0.66 (0.45–0.86) | N/A |
|  | SVM | 51.5 | 44.4 | 54.2 | 0.58 (0.33–0.83) | 0.03* |
|  | LR | 63.6 | 66.7 | 62.5 | 0.63 (0.40–0.84) | 0.11 |
|  | LDA | 63.6 | 66.7 | 62.5 | 0.69 (0.51–0.88) | 0.09 |
|  | KNN | 51.5 | 66.7 | 45.8 | 0.53 (0.35–0.70) | < 0.01** |
|  | RF | 66.7 | 33.3 | 79.2 | 0.63 (0.41–0.84) | 0.15 |
|  | DT | 60.6 | 44.4 | 66.7 | 0.56 (0.36–0.78) | 0.04* |
| Model 3 | Classifier | Accuracy (%) | Sn (%) | Sp (%) | AUC | *P*-value |
|  | CNN | 63.6 | 55.6 | 66.7 | 0.62 (0.36–0.86) | N/A |
|  | SVM | 48.5 | 44.4 | 50.0 | 0.52 (0.26–0.78) | < 0.01** |
|  | LR | 60.6 | 66.7 | 58.3 | 0.59 (0.34–0.85) | 0.03* |
|  | LDA | 60.6 | 66.7 | 58.3 | 0.64 (0.41–0.86) | 0.11 |
|  | KNN | 48.5 | 66.7 | 41.7 | 0.44 (0.23–0.68) | < 0.01** |
|  | RF | 60.6 | 11.1 | 79.2 | 0.57 (0.35–0.78) | 0.10 |
|  | DT | 51.5 | 33.3 | 58.3 | 0.46 (0.27–0.65) | 0.02* |

Abbreviations: Sn, sensitivity; Sp, specificity; AUC, area under the curve; CNN, convolutional neural network; SVM, support vector machine; LR, logistic regression; LDA, linear discriminant analysis; KNN, k-nearest neighbors; RF, random forest; DT, decision tree; N/A, not applicable. **P* < 0.05. ***P* < 0.01. *P­*-values were indicated comparison between convolutional neural network and the other classical models.

**Supplementary Figure S1.**

**SHAP (SHapley Additive exPlanations) Values of Variables Selected for Constructing Each of the Three CNN Models**

Based on the Sharp values, variables such as the indication for PMI, eGFR, IHD, and RV lead position were important for predicting the onset of PICM in all CNN models. On the other hand, factors like LVEF and LBBB, previously identified as risk factors for PICM onset in cohort studies, did not contribute significantly to predicting PICM onset in our analysis.


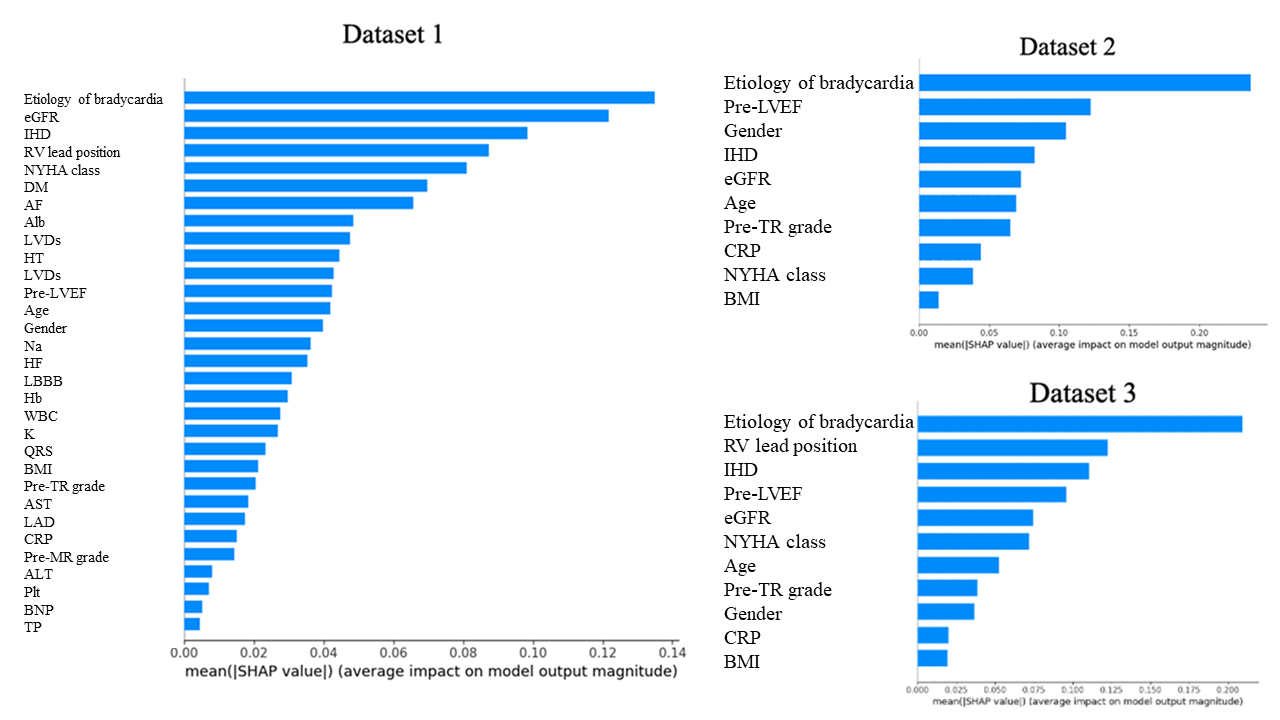


AF, atrial fibrillation; AST, aspartate transaminase; ALT, alanine transaminase; eGFR, estimated glomerular filtration rate; IHD, ischaemic heart disease; LBBB, left bundle branch block; LVEDd, left ventricular end-diastolic diameter; LVEDs, left ventricular end-systolic diameter; LVEF, left ventricular ejection fraction; MR, mitral regurgitation; NYHA, New York Heart Association; PMI, pacemaker implantation; RV, right ventricular; TR, tricuspid regurgitation; Plt, platelet count; WBC, white blood cell count.
